# Assessing altered executive functioning in substance use disorder: Evidence from a novel neurocognitive screening battery

**DOI:** 10.1192/j.eurpsy.2021.1495

**Published:** 2021-08-13

**Authors:** M. Balconi, D. Losasso, A. Balena, D. Crivelli

**Affiliations:** 1 International Research Center For Cognitive Applied Neuroscience - Irccan, Research Unit In Affective And Social Neuroscience, Department Of Psychology, Catholic University of the Sacred Heart, Milan, Italy, Milan, Italy; 2 Serd Canzio, Dsmd, ASST Fatebenefratelli-Sacco, Milan, Italy; 3 International Research Center In Cognitive Applied Neuroscience – Irccan, Research Unit In Affective And Social Neuroscience, Department Of Psychology, Catholic University of the Sacred Heart, Milan, Italy

**Keywords:** Neurocognitive screening, Cognitive control, Substance Use Disorder, Executive functions

## Abstract

**Introduction:**

Recently, clinical models based on neuroscientific evidence have highlighted the detrimental role of executive functions impairments in negatively contributing to the functional decline of patients with Substance Use Disorder (SUD). Yet, despite these potential implications, the screening tools that are typically used to assess such impairments are not specific for patients presenting addiction and are not able to properly sketch their dysfunctional executive control profile.

**Objectives:**

This study aimed at testing the clinical potential of a novel screening battery for neurocognitive disorders in addiction.

**Methods:**

The screening battery was tested on 151 patients with SUD and 55 control subjects. The battery consisted of five neuropsychological tests tapping on verbal and working memory, focused attention, and cognitive flexibility and two computerized neurocognitive tasks (Stroop and Go/No-go tasks adapted for the evaluation of interference inhibition, executive control, and attention bias towards drugs of abuse).

**Results:**

Statistical analyzes showed worse cognitive performance in patients with SUD compared to controls, both at neuropsychological tests of cognitive flexibility, focused attention and verbal memory and at neurocognitive tasks, suggesting the presence of deficit of regulatory mechanisms involved in inhibition and orientation of attention/cognitive resources. These results were also confirmed by second-level analyses where the role of age and education as potential moderators was checked, suggesting the robustness of the tested measures.

**Conclusions:**

The results further stress the link between specific executive impairments and SUD and suggest the potential of the battery as a quick yet valid neurocognitive screening tool.

